# Experiences and Perceptions of Functional Recovery in Late‐Life Depression: A Qualitative Study

**DOI:** 10.1111/inm.70182

**Published:** 2025-12-01

**Authors:** Shannon Cardol, Ilse Timmerman, Jon Matthews, Peter Lucassen, Rose Collard

**Affiliations:** ^1^ Department of Psychiatry Radboud University Medical Center Nijmegen the Netherlands; ^2^ Department of Primary and Community Care Radboud Institute for Health Sciences, Radboudumc Nijmegen the Netherlands

**Keywords:** depressive disorder, functional status, geriatric psychiatry, mental health recovery, qualitative research

## Abstract

This qualitative study aims to explore patient perspectives and experiences with functional recovery in late‐life depression. Purposive sampling was used to include patients (60 years and older) with a depression diagnosis in the past year (in full or partial remission at inclusion). This led to the inclusion of 12 participants. Participants were recruited from an outpatient psychiatric clinic in the Netherlands. Semi‐structured interviews were conducted, transcribed and analysed using both inductive and deductive thematic analysis. The Social Production Function (SPF) theory was utilised as a framework to deductively categorise themes under the subjective well‐being instrumental goals. This study highlights the critical role of stimulation/activation, comfort, affection and aging in functional recovery in late‐life depression. Under the theme of stimulation/activation, we found that motivation, participation, balance and structure were considered highly relevant. Affection, in the form of support, maintaining contact with people and cultivating meaningful connections, made a valuable contribution to the functional recovery process. Within the theme of comfort, participants described how comorbidities affected their recovery, requiring adjustment and acceptance. While themes arose that corresponded to status and behavioural confirmation, they were considered less significant to the recovery process. The inductively found theme ‘the role of aging’ was added to the themes of the SPF framework. It showed that functional recovery from late‐life depression could not be understood without the effects of aging. These results show how the SPF theory aligns with older adults' goal of well‐being. The findings underscore the need for personalised care focusing on functional recovery and offer a foundation for the evaluation of future interventions.

## 1 Introduction

Late‐life depression is significantly associated with impaired functioning (Collard et al. 2018; Wassink‐Vossen et al. 2019; Lenze et al. 2005). Functional impairment can have far‐reaching effects, severely impacting overall well‐being and limiting participation in various aspects of daily life (Sheehan et al. 2017). Even more concerning is that, within 2 years of their initial diagnosis, 50% of older adults with depression experience symptomatic recovery, yet only 20% achieve functional recovery during the same period (Lenze et al. 2005; Collard et al. 2018; Wassink‐Vossen et al. 2019). Moreover, patients often report that maintaining the ability to function in daily life is just as crucial as achieving symptomatic recovery (Bickenbach et al. 2012; Zimmerman et al. 2012). This underlines the need for a deeper understanding of how functional impairments during and after late‐life depression are experienced by older adults so that effective new treatment options can be developed. We used the Social Production Function theory (SPF) (Lindenberg 1986; Ormel et al. 1999) to guide our understanding of how late‐life depressed patients achieve recovery from functional impairment.

## 2 Background

Depression is one of the most prevalent mental disorders worldwide and one of the leading causes of disability among older patients (Horackova et al. 2019; Vos et al. 2020). Depression in older adults is commonly referred to as late‐life depression. Although no official age criterion is set, the term generally applies to those aged 60 and above (Gundersen and Bensadon 2023). In community samples, the prevalence rates of major depressive disorder are between 1% and 4% and in primary care between 6% and 9% (Blazer 2003; Husain‐Krautter and Ellison 2021). Late‐life depression can manifest with a chronic pattern of remission and relapse and has an adverse prognosis with increasing age (Blazer 2003; Schaakxs et al. 2018). Patients with late‐life depression experience higher levels of comorbidity, mortality, cognitive deterioration, dementia and place a higher burden on health care, compared to depressed younger adults (Riddle et al. 2017; Bock et al. 2017; Taylor 2014).

Depression in later life is characterised by depressive symptoms and impairments in daily functioning (i.e., the level of disability). The latter is acknowledged in the Diagnostic and Statistical Manual of Mental Disorders 5th edition (DSM‐5) criteria by including functioning as a key diagnostic criterion (American Psychiatric Association 2013). However, symptomatic recovery does not automatically imply functional recovery (Wassink‐Vossen et al. 2019; Collard et al. 2018; Lenze et al. 2005). Considering this, it is important to use functioning as an outcome in treatment studies for late‐life depression. However, the evidence for treatments targeting functional impairment is limited (Wassink‐Vossen et al. 2022). Only a few randomised controlled trials used functioning as an outcome and applied a multitude of scales of which many were not validated (Wassink‐Vossen et al. 2022; Bingham et al. 2018).

Before adequate treatment options can be developed, it is necessary to enhance our comprehension of the lived experience of functional impairment among older adults during and following late‐life depression. We found one study aiming to understand patients' experiences of impairment in depression in younger adults (Hanson and Young 2017) and one study in late‐life depression (Leibold et al. 2014). The latter focused on coping with functional impairment as well. They both explored patients' experiences with functional impairment during a depressive episode. The results revealed that patients were often hindered by factors such as low physical or mental energy, avoidance, affective symptoms, beliefs that engagement was meaningless, physical pain or a lack of social interaction (Leibold et al. 2014; Hanson and Young 2017). What remains unknown is how participants achieved functional recovery. To our knowledge, no studies have been published to date that specifically explore the experiences and perceptions of functional recovery in older adults with depression.

According to the World Health Organization, the concept of ‘functioning’ encompasses not only bodily functions and physical characteristics, but also what individuals do and who they aspire to be (Bickenbach et al. 2012). The ability to function is closely linked to the capacity of an individual to participate in society. For example, functional impairment can diminish overall well‐being and adversely affect work, social interactions, activities and community participation (Sheehan et al. 2017). To deepen our understanding of functioning in late‐life depression, we used the SPF theory (Lindenberg 1986; Ormel et al. 1999), which has demonstrated its usefulness in studies on well‐being, age‐related phenomena and themes relating to functioning (Gerritsen et al. 2004; Ormel et al. 1997; Bunt et al. 2017). The theory defines functioning as the presence of physical and social well‐being. These are achieved by fulfilling instrumental goals: stimulation/activation, comfort, status, behavioural activation and affection. These goals overlap with the domains in which individuals function.

Therefore, to gain a better understanding of the concept of functional recovery in late‐life depression, we aim to answer the following research question: what are the perceptions and experiences of functional recovery of patients with late‐life depression as seen through the lens of SPF theory?

## 3 Methods

### 3.1 Research Design

A qualitative design was used to gain an in‐depth understanding of specific themes related to recovery, late‐life depression, functional impairment and well‐being. This study was reported according to the consolidated criteria for reporting qualitative research (COREQ) (Tong et al. 2007).

According to the medical ethics committee (CMO Radboudumc), this study does not fall under the jurisdiction of the Medical Research Involving Human Subjects Act (WMO), and a waiver was obtained. All participants signed an informed consent form after having been adequately informed about the study. This study was preregistered in the Center for Open Science (Cardol et al. 2024).

### 3.2 Theoretical Framework: Social Production Function Theory

The Social Production Function (SPF) theory (Ormel et al. 1999; Lindenberg 1986) provides a useful framework to gain in‐depth understanding of experiences with functional recovery in late‐life depression. The use of this framework is supported by Gerritsen et al. (2004), who evaluated frameworks that enhance the understanding of age‐related phenomena. See Table 1 for the complete SPF theory framework. A comprehensive explanation of the SPF theory framework can be found in Appendix S1.

**TABLE 1:** The hierarchy of social production functions, adapted from Ormel et al. (1999).

Top level	Subjective well‐being				
Universal goals	Physical well‐being		Social well‐being		
First‐order instrumental goals	Stimulation/activation (optimal level of arousal)	Comfort (absence of physiological needs; pleasant and safe environment)	Status (control over scarce resources)	Behavioural Confirmation (approval for ‘doing the right things’)	Affection (positive inputs from caring others)
Activities and endowments (means of production for instrumental goals) (examples)	Physical and mental activities producing arousal	Absence of pain, fatigue, thirst, hunger, vitality, good housing, appliances, social welfare, security	Occupation, lifestyle, excellence in sports or work	Compliance with external and internal norms	Intimate ties, offering emotional support
Resources (examples)	Physical and mental effort	Food, health care, money	Education, social class, unique skills	Social skills, competence	Spouse, empathy, attractiveness

### 3.3 Setting

Data were collected from November 2023 until January 2025. The interviews took place either at the outpatient clinic of the Radboud university medical centre (Radboudumc) in the Netherlands, or at the participants' homes.

### 3.4 Participants

Patients were recruited at the psychiatric outpatient clinic of the Radboudumc in the Netherlands and were selected using purposive sampling, taking into account age, gender and somatic comorbidities (retrieved from electronic patient record). Participants were eligible if they were aged ≥ 60 years and had met the DSM‐5 criteria for major depressive disorder at some point during the preceding year, confirmed by the Structured Clinical Interview for DSM‐5 Disorders–Clinician Version (Dutch translation). The depression was in full or partial remission at the time of inclusion, confirmed by a recent score of < 26 (mild or no depressive symptoms) on the Inventory of Depressive Symptomatology‐Self Rated (IDS‐SR) (Rush et al. 1986). In case the IDS score was ≥ 26, participants were still included if the treating clinician observed a clinical improvement. The participants were asked to fill in the IDS‐SR questionnaire before the interview if the previous IDS‐SR score was more than 2 weeks old. Participants were assisted if they were unable to fill in the form by themselves. Exclusion criteria were dementia, bipolar disorder or acute psychosis. Spouses could be present during the interview and were allowed to provide additional comments.

### 3.5 Data Collection

We carried out semi‐structured face‐to‐face interviews with 12 participants. The interviews were recorded with a voice recorder and transcribed verbatim. After conducting 10 interviews, we evaluated saturation, defined as the point at which no new subthemes emerged from the data. To confirm that saturation had been reached, two additional interviews were conducted, and no additional subthemes were identified.

Interview questions were based on the SPF theory and the World Health Organization Disability Assessment Schedule 2.0 (Üstün et al. 2010). The interview guide was piloted by SC and JM with a patient who met all the inclusion criteria except for the age criterion. No adjustments were made to the interview guide. During the analysis cycle, the interview guide was extended with additional questions regarding the themes of comfort, behavioural confirmation, status and age‐related phenomena to achieve a greater understanding of these themes. See Appendices S3 and S4 for the complete interview guide.

The interviews were conducted by SC and JM. Both researchers had basic experience in conducting qualitative interviews and extensive experience with patient communication. Several steps were taken to improve their interview skills. Firstly, they performed a trial interview with a colleague and received feedback to improve their interview skills. Secondly, SC conducted the pilot interview that was observed by JM, with the exchange of feedback afterwards. Lastly, both researchers jointly conducted the first two interviews, alternating between the roles of interviewer and observer. One interview was conducted by RC, who had moderate interviewing experience. All interviews were discussed afterwards.

### 3.6 Data Analyses

Data analysis and data collection were conducted iteratively. The data were analysed using inductive and deductive thematic analysis, according to the six‐stepped approach of Braun and Clarke (2006) We first read and re‐read the interviews (SC and RC or JM). Codes and subsequently subthemes were generated from the transcript using an inductive approach. Following this, themes were generated in a deductive manner using the instrumental goals from the SPF theory. A new theme was established to fit the data if its content was not within the range of the SPF framework. The data were independently analysed by two researchers (SC and RC or JM) using Atlas.ti version 23 and 24 (ATLAS.ti Scientific Software Development GmbH 2023, 2024). Following the separate analysis of each interview, the researchers (SC and RC or JM) compared their codes and discussed them to reach consensus. If there was disagreement, a third researcher (RC or JM) was consulted to facilitate consensus. SC, RC and PL discussed all subthemes and assigned them to the SPF themes to reach consensus on the final categorisation.

All transcripts were written and analysed in Dutch. After choosing relevant quotes, the authors made suggestions for translations. JM (native English speaker) reviewed and finalised the refinement of the quotes to ensure linguistic accuracy.

### 3.7 Trustworthiness

To enhance confirmability a member check was performed on all participants by SC or JM. Member checking involved (dis)agreement by the participant to a verbalised and/or written summary, with the opportunity to provide comments and suggest changes to the summary of the perceived main points. If participants preferred, a full transcript of their interview was provided. All participants received a verbalised summary at the end of the interview. A written summary and full transcript were provided to one participant. Another participant wished to receive only a summary. Full transcripts were provided to seven participants. Two participants wished to receive no written summary or transcript. If the participants desired to change their summary or transcript they could respond to the letter or e‐mail. None of the participants responded which was perceived as an agreement to the summary or transcript (Varpio et al. 2017). Furthermore, to maximise credibility we formed a heterogeneous research team, ensuring researcher triangulation. The multidisciplinary research team consisted of an early career health scientist with a nursing background (SC, MSc, female), a mid‐life experienced mental health nurse practitioner and health scientist (RC, PhD, female), a mental health nurse and early career health scientist (IT, MSc, female), a mid‐life experienced research nurse (JM, PhD, male) and a retired general practitioner and broadly experienced senior researcher (PL, MD, PhD, male). By involving researchers from three different generations and professions in the analysis (SC, RC, JM and PL), we ensured diverse analytical perspectives. Simultaneously, this influenced our reflexivity. To ensure reflexivity we held open discussions with the multidisciplinary research group to interpret the data and facilitate collaboration. The researchers were aware of their own personal beliefs, background and experiences related to this topic (Olmos‐Vega et al. 2023). While two researchers had more scientific and qualitative experience (RC and PL), the other three research team members were relatively early in their qualitative research careers (SC, IT and JM). This diverse experience ensured that the more experienced researchers facilitated strong awareness and methodological robustness whereas the early career researchers provided fresh and open perspectives on the participants' narratives. Additionally, a logbook was kept including changes and decisions made during the study to increase dependability. Moreover, purposive sampling techniques were used to ensure sample heterogeneity, enhancing transferability. All participant quotes were translated by a native English speaker (JM).

## 4 Results

This study included five male and seven female participants (*n* = 12) with ages ranging between 60 and 79 years. The participants had a mean IDS score of 22.3 (12.7). Further sample characteristics are reported in Table 2.

**TABLE 2:** Demographics of Participants *n* = 12.

Demographic characteristics	
Age, mean (SD)	69.0 (6.7)
Age group, %:	
60–69	50.0
≥ 70	50.0
Gender, female, %	58.3
IDS, mean (SD)	22.3 (12.7)

The following themes were described in accordance with the SPF theory: stimulation and activation, comfort, status, behavioural confirmation and affection. Additionally, we found a sixth novel theme, namely the role of aging. This theme intertwines with all SPF goals and plays a pivotal part in the functional recovery process of older adults. The categorisation of the subthemes within the themes is described in Table 3 (below) and Figure S2 (Appendix S2).

**TABLE 3:** Categorisation of subthemes according to SPF framework.

Stimulation/activation	Comfort	Status	Behavioural confirmation	Affection	The role of aging
Balance and structure	(Somatic) comorbidity	(In)dependence	Personality, self‐image, and emotions	Keeping in touch	Cognition
Participation	Adaptation and acceptance	Work		Support and help	Life experience
Motivation and activation	Depression treatment (without pharmacotherapy)			Meaningful relationships and contacts	Future
	Pharmacotherapy				Working and older age
	Mobility				Physical changes and limitations
	Fears and concerns				Loss
	Sleep				
	Depression symptoms				
	Coping mechanisms				
	Recovery				

### 4.1 Stimulation and Activation

Participants experienced a loss of motivation but acknowledged that finding the drive to engage in any activity was beneficial for their recovery. This was described as doing things you would rather avoid but pushing yourself to do them anyway. An example of this was described by a participant who did not consider himself a ‘creatively gifted man’ [observation: ironic undertone] (man, 62 years), but decided it would be helpful to enroll in a creative course at the hospital to keep himself occupied. This approach was further supported:

Yes, I learned last time that sitting and doing nothing will only make you sicker. So try, with whatever little energy you still have, to do something that will take your mind off things. That also helped. So do things that you don't really feel like doing. For example, my husband said, come on, let's go for a walk. And then we went for a walk, even though I really didn't feel like it. And when I came back, I said: yes, that was actually quite nice. So do things anyway, even if you would rather not do them. (woman, 65 years)

Participants indicated that some kind of external influence was necessary to spark enough motivation to engage in various activities. These influences included societal pressure, expectations from employers, financial concerns, advice from caregivers and pressure from spouses or family members. Spouses played a crucial role in motivating participants during their functional recovery. Additionally, participants found that taking care of a dog provided strong motivation to stay active or even simply get out of bed. Participants, even those who did not own a dog, believed that having one could be beneficial in their functional recovery process.

I suddenly think, what if you had a dog that knew to pull the duvet off you in the morning. (…) That would be a nice job for a service dog. (woman, 63 year)

Although undertaking activities in any form was considered crucial, energy levels did not always allow participants to be active throughout the day. Another challenge participants encountered was avoiding doing too much.

Now I can dose it better and I have to say that it's getting better step by step. Before that, I was very nervous, tense and kept very busy. I did this, I did that and then you expect a pat on the back from yourself saying, well, you're doing well. But that simply didn't work. So yes, I really had to put the brakes on. (woman, 75 years)

In addition to the balancing of energy levels and activities throughout the day, having a specific routine was also helpful in achieving activation. For instance, participants attended appointments or did grocery shopping.

What I sometimes do now is that I uh, is that I set an alarm clock if I think I have to get out of bed at a certain time. Then I set an alarm clock, but I put it in the hallway and then it goes off. But it gets very annoying at a certain point because it keeps going off and then you have to, to turn off that sound, you have to get out. (woman, 63 year)

### 4.2 Comfort

Depression often brings a range of discomforting symptoms, such as sadness, fatigue, stress and pain. However, the participants we interviewed experienced not only the effects of depression but also the challenges posed by comorbid somatic conditions, such as Parkinson's disease or arthrosis.

It's harder to get out of bed in the morning and get moving. But that's also partly because the Parkinson doesn't really help, you know. (…) And getting yourself started for the day is now difficult due to Parkinson's. But the depression doubles the problem. (woman, 63 years)

I really enjoy working in the garden, but because of the disability with my hip and my knee and everything, that no longer happens. (…) I also enjoy walking the dog. But that's not going as well, uh, as well as I would like. (woman, 75 years)

Participants mentioned that it is important to accept that things have changed and adjust to deal with depression and its challenges. Examples were shared by participants regarding being unable to perform their hobbies at their desired level and how they coped with that:

I can do a lot less with my body. Uh, I also found that annoying, of course. (…) I used to have a certain level. (…) So, things didn't go the way I wanted. I also had to come to terms with the fact that it just wasn't possible anymore, so to speak. So, I signed up to play tennis with older ladies. (…) But it's hard when you are used to a certain level, and then suddenly you're playing tennis at a much lower level. I really noticed that the difference was quite big. (woman, 61 years)

Accepting change was just as important. Participants explained they had to quit their hobbies due to physical impairments and had to accept the fact that they were not able to do perform their usual activities.

To manage psychological impairments, participants described beneficial nonpharmacological treatments such as psychotherapy.

I first, uh, that was on the advice of the doctor. That I had to meet others with the same condition. (…) And then I was in a group, and they were all depressed. And all depressed in different ways. (…) And then I was given homework. And then we had to, we weren't allowed to talk negatively. You had to convert all the sentences that were negative into positivity. And I thought that then…, well I shouldn't exaggerate. But in retrospect, I think that it may have helped after all. (woman, 75 years)

It was less helpful for others. We found that cognitive behavioural therapy was not the right match for everyone.

I felt a bit like I was back in school. That I had to do things again. I liked hearing things, but I didn't really like the fact that you had to do it yourself as part of an exercise. (…) because I didn't understand everything there and then he had to explain it a bit again. I always felt very insecure about it. Like, am I doing it right? That school feeling. I didn't like that. (woman, 65 years)

Participants took antidepressants and were satisfied with their medication, believing it had helped them to recover. Participants articulated the belief that they would not have recovered without them.

I used to take another medicine. (…) at a certain point it didn't work for me anymore, so the psychiatrist advised a switch to the medication I'm taking now. (…) and that works for me. (man, 62 years)

### 4.3 Status

Participants described forms of achieving, retaining, or losing status. Of those working, participants were not able to perform their jobs as usual, had to do customised tasks, had to take sick leave or were even declared unfit to work. Although the latter led to feelings of insecurity at first, eventually participants were quite relieved they could stop working.

I've been to the company doctor, and he said, ‘there are so many women suffering with much less than you who have stopped working. Go and enjoy your grandchildren’, he said. ‘And just tell yourself, I'm taking early retirement’, he said. (…) so now I have found peace in that too. (woman, 61 years)

We also found that unemployment did not necessarily result in a loss of status. Being an informal caregiver for family, a handyman, volunteer or working in the garden were additional ways through which participants sought to regain the sense of fulfilment they previously found in paid work.

Status can also be linked to autonomy. Participants highlighted the importance of self‐management, sharing examples of maintaining control over decisions such as which activities to pursue, what to avoid, or where to live.

(Being independent is important because) then you can decide for yourself what you are going to do or what you are not going to do. What you can or what you can't do. Yes, of course, you want to be in control of that for as long as possible. (man, 62 years)

### 4.4 Behavioural Confirmation

When individuals felt they did not act in line with expectations, this could lead to feelings of shame or insecurity. Participants expressed concerns about their cognitive abilities, sharing experiences where they felt their communication was unclear or when they had to admit not understanding everything. There were also instances in the workplace where participants believed they had to live up to certain norms:

In 2000 I was diagnosed with breast cancer with metastases. Yes, I had a few surgeries during that period, so I was often sick. Because things weren't going well at work, I even underwent the last surgery during my vacation. I thought I'd get it done during my vacation because I thought, uh, I was afraid to say anything at work. It really bothered me. And that was really uh very difficult. (woman, 61 years)

On the other hand, feelings of pride also emerged by driving a car again or a sense of self‐worth by helping others.

Yes, I think… uh, yes, well, that I can get more involved again—for others. For the children, and, well, for my mother. And in other areas too, actually. Yes, just being able to make myself useful again. If I may say so, that really gives me more self‐esteem—that I can show I'm able to do that, and that I've actually done it. (man, 60 years)

### 4.5 Affection

Participants emphasised that support and care from others were critical to their functional recovery process. We found the important role of contact with others and the presence of meaningful relationships in their recovery. In many cases, practical assistance was provided including being driven to appointments, cooking and domestic help. Additionally, emotional support was readily available.

A friend came. (…) She came to get me. (…) We left and were two streets away when I said ‘oh, my back hurts. I'm going back home’. She said ‘What are you thinking? I didn't come to you to stop after fifteen minutes! No, we'll keep going’. (woman, 75 years)

Despite this, participants mentioned not wanting to burden others or a lack of support from loved ones resulted in feelings of anger.

But sometimes he (participants' husband, eds.) feels like that, sometimes things take too long and then he can sometimes say, how is that possible? Then I think, get lost! (…), that always makes me very angry. (woman, 76 years)

Breaking social isolation was part of the recovery process for participants.

I simply sent people an email or called them again. And then I said like ‘Can we make an appointment?’ Or, for example, if it was someone's birthday. That's always so easy, isn't it? You can send a card. A birthday card. And then you write in the card: when shall we meet? When are we going to have coffee? (woman, 75 years)

An important topic for participants was feeling heard or understood, by for example their friends, healthcare provider or employer. Conversely, participants also described a misunderstanding of mental health issues.

Well, I sometimes didn't understand it when someone close to me had depression and I hadn't experienced depression at that point. And then I thought, wow. She should be happy. She has all this and all that. And now uh a depression? You need a kick up the backside! That was what I thought. (…) then afterwards, when I had that depression, I thought how could I have ever thought like that? And that is of course the very dangerous thing about having depression, getting depression. (…) Yes, getting something and understanding that someone else cannot relate to it. And that the other – the other person can't be blamed for that either. Just be glad that they can't relate to it… can you imagine? Then they'd have it too! (woman, 75 years)

I think that it's worse being mentally ill than having something physical. A friend recently died of cancer, and I always said I wish I had cancer. (…) that's bad too, I'm not saying it isn't. But (…) people deal with that more easily. And (…) when it comes to mental illnesses, they say ‘don't worry about it’ or ‘there is nothing to be afraid of’. I could punch them (when they said that). (…) They have no idea how it all feels. (woman, 76 years)

### 4.6 The Role of Aging

Aging often comes with a range of challenges, including the loss of loved ones, uncertainty about the future and physical and cognitive impairments. Participants referred to the aging process suggesting that functional recovery from late‐life depression cannot be understood without considering the role of aging.

Participants noted that aging and depression often led to cognitive impairments, complicating functional recovery. These impairments were primarily experienced as memory complaints.

I get you a glass of water and I pour it and I sit down again and then I forget to bring the drink. (…) Or I set the table and then I don't put down any plates and then my wife says you forgot the plates again. (man, 62 years)

I didn't know the way home anymore. I drove to my daughter where I drove a hundred times. I drove around. I thought I didn't know how to get home anymore. (woman, 61 years)

The loss of loved ones such us the death of a family member, friends moving to nursing homes or neighbours becoming ill was also challenging:

But uh yes, when you're older, people die, your friends leave, there's very little left. So that's also a difficult time that you have to get through. (woman, 75 years)

Participants felt they were not able to do the same things as when they were younger. This was described as:

Yes, getting older and not being able to do anything anymore. You know, that didn't really play a role at that time. I was physically able but just didn't do things. And now I can't do things in any case. (woman, 75 years)

However, aging can also provide certain benefits such as life experiences that may help the recovery process:

Aging means that you have experience with depression, for example. That you've learned how to deal with it in the best way. So yes, your life experience does of course help you in that sense. (woman, 63 years)

## 5 Discussion

The focus of this study was to explore the experiences and perceptions of older adults with late‐life depression of their functional recovery process through the lens of SPF theory which proved a valuable framework, as all instrumental goals were described by the participants. The following goals of the SPF theory featured more prominently: stimulation/activation, comfort and affection, while status and behavioural confirmation were emphasised less. We added the theme of the role of aging to describe the full experience of functional recovery in late‐life depression.

Stimulation and activation encompassed motivation to participate in activities, as well as structure and balance in daily routines. This reflects the SPF theory as it describes how people undertake mental and physical activities that produce arousal. Quantitative studies have demonstrated that motivational deficits, often present as residual symptoms after antidepressant treatment, negatively influence the relationship between depression and functioning, as well as subjective well‐being (Fervaha et al. 2016; Raskin et al. 2012). This stresses the importance of including motivation in effective treatments for functional impairment in depression (Calabrese et al. 2014).

In our study, the instrumental goal of affection was highly valued by participants. These findings align with the well‐documented link between depression and loneliness, as depression is often exacerbated or triggered by social isolation (Kawachi and Berkman 2001). Furthermore, qualitative research emphasises the critical role of social interactions throughout the course of depressive episodes—before, during and after their occurrence. Supporting this perspective, Nair et al. (2022) identified socialising and informal support as key elements in the self‐management of depression among frail older adults.

Comfort emerged as essential to achieving functional recovery. We identified several factors supporting this goal. Notably, participants expressed satisfaction with their antidepressant medication, which was somewhat surprising. While qualitative studies often report mixed results on antidepressant satisfaction, they generally conclude patients' experiences with antidepressants are complex and evolve over time, encompassing both positive and negative aspects (Gibson et al. 2014). Depression is typically first treated within a primary care setting in the Netherlands. If first‐step interventions prove ineffective or too complex, patients are referred to secondary care, specifically specialised mental health services (van Avendonk et al. 2012). Participants in this study had passed the stage of primary care treatment and might not have benefited sufficiently from treatment in this setting. This may have led to a more favourable view of medication. Moreover, the participants' experiences with dealing with comorbidities during the functional recovery process underscore the need for a more holistic approach to treating late‐life depression. Addressing both mental and physical health challenges is crucial to enhancing functional recovery outcomes.

Topics supporting status were described by the participants; however to a lesser extent than the other instrumental SPF goals. This may be because social well‐being, consistent with the SPF theory, was adequately addressed through other goals suggesting that substitution of goals may lead to sufficient well‐being without achievement of all goals (Ormel et al. 1999; Lindenberg 1986). Status, as described by Lindenberg (1986) and later Ormel et al. (1999) has some overlap with self‐actualisation described by Maslow (1987). Whereas status is heavily influenced by external and social evaluation of others, self‐actualisation focuses more on personal fulfilment, independent of social validation. An explanation could be that the participants have risen above these social constructs and increasingly rely on their intrinsic feeling of self‐worth.

Topics linked with behavioural confirmation were also less frequently mentioned. Behavioural confirmation refers to the process by which individuals' actions are shaped by their perceptions of others' expectations. This phenomenon can present as stigma, stereotyping, adherence to societal norms and values or self‐fulfilling prophecy. Behavioural confirmation in the context of depression is commonly conceptualised as an expression of dysfunctional social expectations, which is also associated with higher depression severity, as well as lower well‐being (Kirchner et al. 2024). The sample analysed experienced low to moderate depressive symptoms which may explain why topics regarding behavioural confirmation were hardly mentioned. Behavioural confirmation is partly driven by the feeling of belonging to a group with shared values. According to Cappelli et al. (2020) a lack of this feeling can lead to social vulnerability and act as a mediator towards functional impairment. It may be that participants had a strong intrinsic sense of belonging or were fulfilled with respect to other social well‐being goals that reduced their reliance on behavioural confirmation.

The last theme was ‘the role of aging’ and was not retrieved from the SPF framework. The articulated benefits of life experience to the recovery process are reflected in the theory of successful aging (Troutman‐Jordan 2015) and the life span theory in developmental psychology (Baltes et al. 2006). Participants also mentioned less favourable circumstances associated with aging, such as loss of loved ones. Encounters with bereavement and grief are common situations as age progresses (Bonanno 2004; Zoler 2006; Treml et al. 2022). Correspondingly, social networks become smaller which can affect the intensity of grief (Carstensen 1992). Other challenges in later life were dealing with cognitive and physical impairments, and uncertainty for the future. A systematic review and meta‐ethnography by Rosenwohl‐Mack et al. (2020) found that individuals aging in their own homes undertook considerable efforts to manage unpredictable needs and challenges. However, when the challenges of aging exceeded their control, the resulting effects often involved feelings of uncertainty, isolation and disconnection (Rosenwohl‐Mack et al. 2020).

This qualitative study involving older adults in the recovery phase of severe depression is both unique and relevant. The use of the SPF theory provided critical insights into the recovery process. We used multiple strategies to strengthen trustworthiness, including all components: credibility, transferability, dependability and confirmability (Ahmed 2024). We interviewed 12 participants, and after the third interview, no new subthemes emerged, while the previously defined subthemes continued to support the new data.

A limitation of this study was the inclusion of participants aged 60 to 79 years. Recruitment of participants aged 80 years and older was challenging because this age group is rarely referred to academic hospitals and is typically treated by general practitioners. High mortality, morbidity and cognitive comorbidities in late‐life depression (Haigh et al. 2018) likely contributed to the difficulty in identifying eligible participants that were 80 years and older. This might have led to missing themes that are valuable for older adults in this age group. Therefore, we recommend that this age group be investigated specifically to gain more understanding of the functional recovery process in the oldest old with depression. Another limitation regarding the transferability of this study is the absence of data on participants' socio‐economic and educational backgrounds. Therefore, we were unable to link participant characteristics to theme differences. Future research would benefit from including background information to enhance the transferability of the findings.

## 6 Conclusions

This study emphasises the critical role of functional recovery in late‐life depression and demonstrates how the SPF theory supports older adults' universal goal of achieving well‐being through functional recovery. Key goals—activation, comfort and affection—emerged as essential targets in the recovery process and highlighted the need for more holistic, person‐centered care, addressing functional recovery in late‐life depression.

### 6.1 Relevance for Clinical Practice

Our findings form a meaningful foundation for the development of nursing interventions using, for instance, a collaborative care approach and the content of the themes within the SPF framework. Since reducing functional limitations is an everyday nursing care goal, nurses are well‐equipped for caring for depressed, functionally impaired patients. Nurses can also directly use the content of the themes in daily clinical practice. This might involve discussion of the key findings, stimulation and activation, comfort, affection and the role of aging and offering assistance in achieving these goals of well‐being, facilitating functional recovery. More specifically, nurses can integrate these goals into individual care plans, thereby promoting a structured and person‐centred approach to functional recovery in late‐life depression. Continued efforts are necessary to develop nursing interventions targeting functional impairment in late‐life depression.

## Author Contributions

Shannon Cardol and Rose Collard were involved in study concept and design. Shannon Cardol, Jon Matthews and Rose Collard were involved in acquisition of data. Shannon Cardol, Rose Collard, Peter Lucassen and Jon Matthews were involved in analysis and interpretation of data. Shannon Cardol and Rose Collard were involved in drafting of the manuscript. Shannon Cardol, Rose Collard, Peter Lucassen, Jon Matthews and Ilse Timmerman were involved in critical revision of the manuscript for important intellectual content.

## Funding

This work was supported by ZonMw, 80‐86300‐98‐123.

## Ethics Statement

According to the medical ethics committee (CMO Radboudumc), this study does not fall under the jurisdiction of the Medical Research Involving Human Subjects Act (WMO), and a waiver was obtained.

## Consent

All participants signed an informed consent form after having been adequately informed about the study.

## Conflicts of Interest

The authors declare no conflicts of interest.

## Supporting information


**Appendix S1:** Social Production Function Theory.

**Appendix S2:** Coding tree.

**Appendix S3:** Interview guide GOLLD‐NP (Dutch).

**Appendix S4:** Interview guide GOLLD‐NP (English translation).

## Data Availability

The data that support the findings of this study are available on request from the corresponding author. The data are not publicly available due to privacy or ethical restrictions.
