# Supplementary material for: Experiences and Perceptions of Functional Recovery in Late‐Life Depression: A Qualitative Study
Source: Int J Ment Health Nurs. 2025 Dec 1;34(6):e70182. doi: 10.1111/inm.70182 (PMC12669942; doi:10.1111/inm.70182)
Supplement: Supplementary file 1 — Appendix S1: Social Production Function Theory. [file INM-34-0-s004.docx]

**Supplement 1. Social Production Function Theory**

According to the SPF theory, people strive for subjective well-being, which has two aspects, namely physical and social well-being. Physical well-being consists of sufficient levels of stimulation/activation and comfort. Stimulation/activation refers to the optimal level of arousal generated by mental and physical activities. Comfort is explained as a physical and mental state in which, for instance pain, stress, and hunger are absent. Social well-being consists of status, behavioural confirmation, and affection. Status refers to a person's position in relation to others, largely determined by their access to and control over valuable or limited resources. Behavioural confirmation is the sense of having acted appropriately or in line with expectations, based on how others perceive you, even in the absence of direct feedback. Affection encompasses “love, friendship, and emotional support and is provided through caring relationships” (Ormel et al., 1999). These 5 factors can be considered as goals to which people aspire (instrumental goals) and are accomplished by participating in activities, obtaining endowments, and using resources. In SPF theory, an individual may substitute a certain goal for another if it satisfies a similar need for that individual.

| Top level | Subjective Well-being | | | | |
| --- | --- | --- | --- | --- | --- |
| Universal goals | Physical Well-being | | Social Well-being | | |
| First-order instrumental goals | Stimulation/activation (optimal level of arousal) | Comfort (absence of physiological needs; pleasant and safe environment) | Status (control over scarce resources) | Behavioural Confirmation (approval for “doing the right things”) | Affection (positive inputs from caring others) |
| Activities and endowments (means of production for instrumental goals) (examples) | Physical and mental activities producing arousal | Absence of pain, fatigue, thirst, hunger, vitality, good housing, appliances, social welfare, security | Occupation, lifestyle, excellence in sports or work | Compliance with external and internal norms | Intimate ties, offering emotional support |
| Resources (examples) | Physical and mental effort | Food, health care, money | Education, social class, unique skills | Social skills, competence | Spouse, empathy, attractiveness |

**Figure S1.** The Hierarchy of Social Production Functions, adapted from Ormel et al. (1999).
