# Supplementary material for: Experiences and Perceptions of Functional Recovery in Late‐Life Depression: A Qualitative Study
Source: Int J Ment Health Nurs. 2025 Dec 1;34(6):e70182. doi: 10.1111/inm.70182 (PMC12669942; doi:10.1111/inm.70182)
Supplement: Supplementary file 2 — Appendix S2: Coding tree. [file INM-34-0-s001.docx]

**Supplement 2. Coding tree**

**Figure S2.** Coding Tree
