# Supplementary material for: Experiences and Perceptions of Functional Recovery in Late‐Life Depression: A Qualitative Study
Source: Int J Ment Health Nurs. 2025 Dec 1;34(6):e70182. doi: 10.1111/inm.70182 (PMC12669942; doi:10.1111/inm.70182)
Supplement: Supplementary file 3 — Appendix S3: Interview guide GOLLD‐NP (Dutch). [file INM-34-0-s003.docx]

**Supplement 3. Interview guide GOLLD-NP (Dutch)**

Introductie

Stel jezelf voor en geef aan dat het interview ongeveer een uur zal duren, opgenomen wordt en geanonimiseerd wordt uitgetypt. Spreek waardering uit voor deelname aan het onderzoek en benoem nogmaals dat deelname vrijwillig is en dat de deelnemer zich op elk moment, zonder opgave van reden, terug kan trekken uit het onderzoek. Vraag vooraf of er nog vragen zijn over de informatiefolder.

Informatie over het onderzoek

Met behulp van dit interview willen we erachter komen welke thema’s belangrijk waren voor u bij het herstel van uw depressie. Dit helpt ons bij het ontwikkelen van een nieuwe behandeling voor beter herstel van depressie op latere leeftijd. Daarbij richten we ons op het herstel van de dagelijkse bezigheden. Herstel kan inhouden dat iemand geen symptomen meer heeft maar kan dus ook op andere gebieden zijn. We zien dat mensen op latere leeftijd vaak minder goed en snel herstellen bij het oppakken van de dagelijkse dingen die voor hen belangrijk zijn. Dus ik ga u vooral vragen stellen over wat u hielp bij het weer kunnen doen van de dagelijkse bezigheden die voor u belangrijk zijn. Dit kan bijvoorbeeld zijn: het weer oppakken van sociale contacten, hobby’s uitoefenen, bewegen, vrijwilligerswerk etc.

Toestemming

Vraag toestemming voor opname en laat deelnemer de toestemmingsverklaring doornemen en ondertekenen.

Start

Voordat we het gaan hebben over uw herstel van de dagelijkse bezigheden, zou ik graag willen weten:

**Hoe is het de afgelopen tijd met u gegaan?**

- Hoe is het beloop van uw depressieve klachten geweest?
- Op welk punt bent u in uw herstel?

**(Uitleggen wat functioneren/functioneel herstel is.) Functioneren gaat over de dagelijkse dingen die mensen doen.**

- **Welke dingen doet u graag, of zou u graag weer willen doen?**
- **Wat is belangrijk voor u?**
- **(Wat doet u zoal overdag?)**

**Hoe werden uw dagelijkse bezigheden beïnvloedt door de depressieve klachten?**

- Op het gebied van:
  - Cognitie
  - Mobiliteit

Alleen stellen wanneer mensen het moeilijk vinden dit concreet te maken

- - Zelfverzorging
  - Omgaan met mensen
  - Activiteiten
  - Participatie
  - Slapen

**Wat was voor u belangrijk in het herstel van de dagelijkse bezigheden?**

- Hoe ging u daarmee om?
- Welke moeilijkheden ervaarde u? Waar zou dat door kunnen komen?
- Aan welke ondersteuning had u behoefte? Hoe zag/ziet dit eruit?
- Welke doelen/wensen heeft u op dit moment?
- Welke keuzes moest u maken?

**Hoe belangrijk was het voor u om dingen te blijven doen/activiteiten te ondernemen?**

**Welke rol speelde motivatie bij het herstel?**

**Wat betekent het voor u om onafhankelijk te blijven?**

**Welke rol speelt uw levensfase/leeftijd in uw herstel?**

- Welke behoeften zijn hierdoor belangrijker of minder belangrijker geworden?

**Samenvatting geven van de meest belangrijke thema’s en vragen of dit klopt.**

**Zijn er nog andere zaken, die niet aan bod zijn gekomen, die u nog belangrijk vindt om te benoemen voor ons onderzoek?**

Doorvragen (hoe, wie, wat, waar, wanneer):

- vraag om voorbeelden, situaties te noemen.
- kunt u daar wat meer over vertellen?
- kunt u daar een voorbeeld van geven?
- Kunt u meer vertellen over u ervaringen daarmee?

Afsluiting

Bedank de deelnemer voor het interview. Geef een samenvatting en vraag of dit overeenkomt met de deelnemers opvattingen. Vraag of de deelnemer op de hoogte gehouden wil worden van de resultaten van het onderzoek. Vertel iets over eindproduct.

**Ter verdieping van al verzamelde data:**

**Comfort**

- Is uw mening tav medicatie/antidepressiva veranderd door het doormaken van een depressie?
- Op welke manier speelde acceptatie een rol in uw herstel? (Kunt u omschrijven hoe u leerde om de nieuwe situatie te accepteren?)

**Behavioural confirmation**

- Welke rol speelt het goed willen doen in de ogen van andere mensen een rol bij u?
- Waar bent u onzeker over?
- Waar bent u trots op?

**Status (misschien kort inleiden door status uit te leggen)**

- Wat doet u om eigenheid te behouden? Is dat belangrijk voor u?
- Op welke manier onderscheidt u zich van anderen?
- Hoe kijkt u tegen pensioneren aan?

**Ouder worden**

- Welke rol speelt ouder worden in het herstelproces?
- Welke positieve ervaringen zijn er gerelateerd aan ouder worden?
