# Supplementary material for: Experiences and Perceptions of Functional Recovery in Late‐Life Depression: A Qualitative Study
Source: Int J Ment Health Nurs. 2025 Dec 1;34(6):e70182. doi: 10.1111/inm.70182 (PMC12669942; doi:10.1111/inm.70182)
Supplement: Supplementary file 4 — Appendix S4: Interview guide GOLLD‐NP (English translation). [file INM-34-0-s002.docx]

**Supplement 4. Interview guide GOLLD-NP (English translation)**

Introduction

Introduce yourself and indicate that the interview will last about an hour, will be recorded and typed out anonymously. Express appreciation for participating in the study and reiterate that participation is voluntary and that the participant can withdraw from the study at any time, without giving a reason. Ask in advance if there are any questions about the information leaflet.

Study information

With the help of this interview, we want to find out what themes were important to you in your recovery from depression. This will help us develop a new treatment for better recovery from depression in later life. In doing so, we focus on recovery from daily activities. Recovery may involve a person no longer having symptoms but can therefore be in other areas. We see that people in later life often recover less well and quickly in picking up the daily things that are important to them. So I am mainly going to ask you questions about what helped you in being able to do the daily activities that are important to you again. This could include picking up social contacts again, pursuing hobbies, exercise, volunteering, etc.

Consent

Get consent for admission and have participant review and sign the consent form.

Start

Before we start talking about your recovery from daily activities, I would like to know:

**How have you been doing recently?**

- How has the course of your depressive symptoms been?
- At what point are you in your recovery?

**(Explain what functioning/functional recovery is.) Functioning is about the daily things people do.**

- **What things do you like to do, or would like to do again?**
- **What is important to you?**
- **(What do you do during the day?)**

**How were your daily activities affected by the depressive symptoms?**

- In terms of:
  - Cognition
  - Mobility
  - Self-care

Only ask when people find it difficult to make this concrete.

- - Dealing with people
  - Activities
  - Participation
  - Sleep

**What was important to you in the recovery of daily activities?**

- How did you cope with this?
- What difficulties did you experience? What could this be due to?
- What support did you need? What did this look like?
- What goals/desires do you have right now?
- What choices did you have to make?

**How important was it for you to keep doing things/activities?**

**What role did motivation play in recovery?**

**What does it mean to you to remain independent?**

**What role does your stage of life/age play in your recovery?**

- What needs did this make more or less important?

**Summarise the most important themes and ask if this is true.**

**Are there any other issues, not covered, that you think are still important to mention for our research?**

Continuing questions (how, who, what, where, when):

- ask to cite examples, situations.
- can you tell us more about them?
- can you give an example of that?
- Can you tell more about your experiences with that?

Closing

Thank the participant for the interview. Give a summary and ask if this matches the participant's views. Ask if the participant wants to be kept informed about the results of the research. Tell something about final product.

**To deepen already collected data (added 13-1-2025):**

**Comfort**

- Has your opinion on medication/antidepressants changed as a result of going through depression?
- In what way did acceptance play a role in your recovery? (Can you describe how you learned to accept the new situation?)

**Behavioural confirmation**

- What role does wanting to do well in other people's eyes play for you?
- What are you insecure about?
- What are you proud of?

**Status (perhaps introduce briefly by explaining status)**

- What do you do to maintain individuality? Is that important to you?
- In what ways do you differentiate yourself from others?
- How do you view retirement?

**Growing older**

- What role does ageing play in the recovery process?
- What positive experiences are related to getting older?
